# Complete genome sequence of an *agr*-dysfunctional variant of the ST239 lineage of the methicillin-resistant *Staphylococcus aureus* strain GV69 from Brazil

**DOI:** 10.1186/s40793-016-0154-x

**Published:** 2016-05-04

**Authors:** Ana M. N. Botelho, Maiana O. C. Costa, Cristiana O. Beltrame, Fabienne A. Ferreira, Marina F. Côrtes, Paula T. Bandeira, Nicholas C. B. Lima, Rangel C. Souza, Luiz G. P. Almeida, Ana T. R. Vasconcelos, Marisa F. Nicolás, Agnes M. S. Figueiredo

**Affiliations:** Laboratório de Biologia Molecular de Bactérias, Instituto de Microbiologia Paulo de Góes, Universidade Federal do Rio de Janeiro, Rio de Janeiro, 21941-902 RJ Brazil; Laboratório Nacional de Computação Científica, Petrópolis, 25651-075 RJ Brazil

**Keywords:** Complete genome sequence, Methicillin-resistant *Staphylococcus aureus*, ST239, Skin hospital infection, *agr* dysfunction

## Abstract

*Staphylococcus aureus* is a versatile Gram-positive coccus frequently found colonizing the skin and nasal membranes of humans. The acquisition of the staphylococcal cassette chromosome *mec* was a major milestone in the evolutionary path of methicillin-resistant *S. aureus*. This genetic element carries the *mecA* gene, the main determinant of methicillin resistance. MRSA is involved in a plethora of opportunistic infectious diseases. The accessory gene regulator is the major *S. aureus* quorum sensing system, playing an important role in staphylococcal virulence, including the development of biofilms. We report the complete genome sequence (NCBI BioProject ID: PRJNA264181) of the methicillin-resistant *S. aureus* strain GV69 (= CMVRS P4521), a variant of the ST239 lineage that presents with a natural attenuation of *agr*-RNAIII transcription and a moderate accumulation of biofilm.

**Electronic supplementary material:**

The online version of this article (doi:10.1186/s40793-016-0154-x) contains supplementary material, which is available to authorized users.

## Introduction

*Staphylococcus aureus* is an adaptable pathogen capable of infecting nearly all tissues and organs of the human body. Methicillin-resistant *S. aureus* is a major bacterial pathogen in terms of its incidence and the severity of associated illnesses. MRSA infections can affect either hospitalized patients or healthy individuals within the community [1]. Hospital-associated MRSA show a highly clonal population, and clonality have usually been characterized based on pulsed-field gel electrophoresis analysis, SCC*mec* typing and multilocus sequence typing. One of the most globally disseminated HA-MRSA lineages is the ST239-SCC*mec*III [1].

We previously reported the complete genome sequence of the ST239 strain BMB9393 from Brazil that expresses high levels of *agr*-RNAIII transcripts, and has a superior ability to accumulate *ica*-independent biofilm [2]. The accessory gene regulator operon is the main quorum-sensing system of *S. aureus*. It is well-known that *agr* regulates a plethora of virulence factors and key mechanisms associated with the pathogenesis of *S. aureus* infections, including the development of biofilm [3]. The *agr*-RNAIII transcripts and the AgrA protein are the regulatory molecules (effectors) of the *agr* operon [4].

We report here the complete genome sequence of an ST239 variant, strain GV69, which has a natural attenuation of the *agr*-*rnaIII* gene expression and forms a thinner biofilm layer in comparison to BMB9393.

## Organism information

### Classification and features

**Fig. 1:**
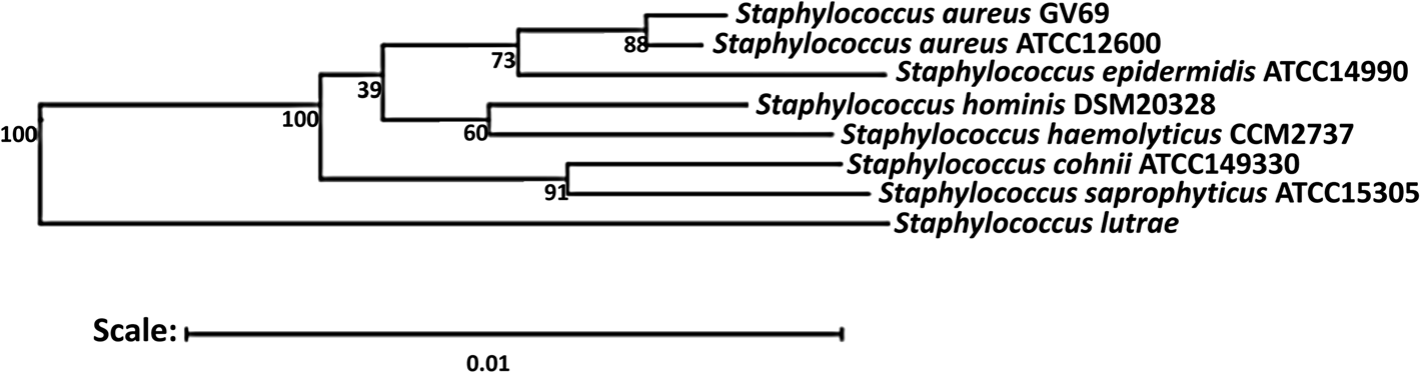
Phylogenetic tree showing the position of the GV69 strain relative to other type strains within the *Staphylococcaceae* family. The strains and their corresponding GenBank accession numbers (in parentheses) for the 16S rRNA genes are as follows: *S. aureus* strain ATCC 12600 (L36472), *S. saprophyticus* strain ATCC 15305 (AP008934), *S. epidermidis* strain ATCC 14990 (D83363), strain DSM 20328 (X66101), *S. haemolyticus* strain CCM2737 (X66100), *S. cohnii* strain ATCC 49330 (AB009936), with (X84731) as an outgroup. To construct the tree, the sequences were aligned with the RDP aligner using the Jukes-Cantor corrected-distance model for assembling a distance matrix based on the alignment model positions without the use of alignment inserts and with a minimum comparable position of 200. The tree was built with RDP Tree Builder, which uses Weighbor with an alphabet size of 4 and size length of 1000 [31]. The bootstrapping process was repeated 100 times to generate a strict consensus tree [32]

**Fig. 2:**
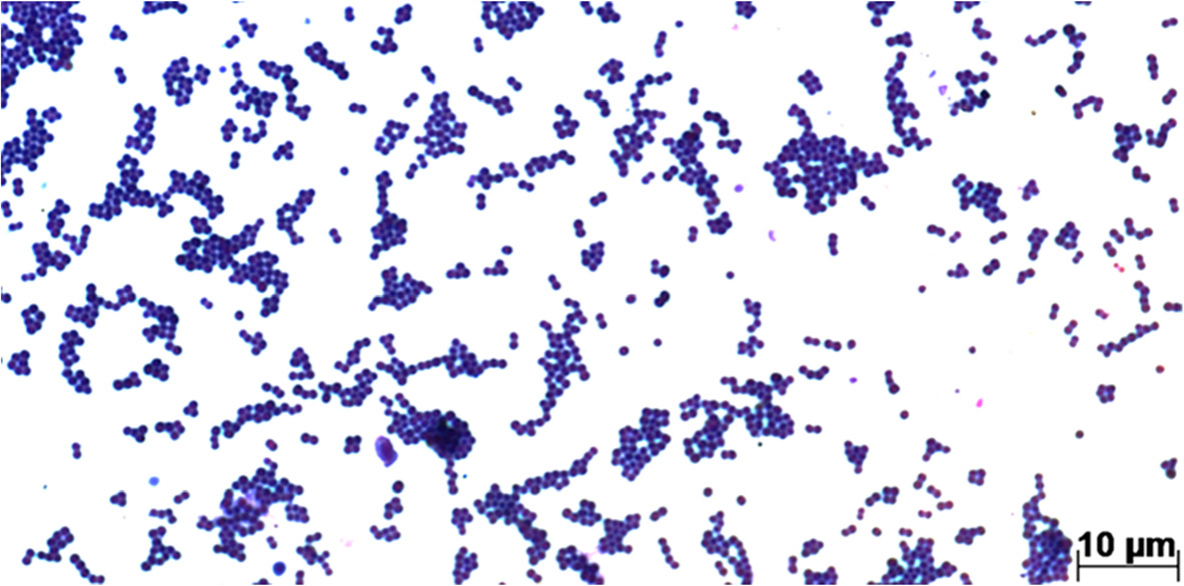
Photomicrograph of the *S. aureus* strain GV69 using Gran stain. bar = 10 μm

We sequenced the complete genome of a variant of the ST239 MRSA lineage called GV69. This strain was isolated in 1996 from a skin wound infection in a patient admitted at a burn unit in a general public hospital in Teresina city, located at the northeast region of Brazil [5]. In Brazil, ST239 isolates are only associated with hospital infections, and they are broadly disseminated, multiresistant, and frequently grouped in the Brazilian epidemic clone, based on PFGE analysis, MLST, and *mec* typing [6–8]. Strain GV69 has a natural *agr* dysfunction and a moderate biofilm phenotype. The ability of many bacteria to develop biofilm is considered an important mechanism of colonization, primarily in infections associated with the use of indwelling medical devices [6]. GV69 strain is a non-motile, non-spore forming, non-hemolytic Gram-positive cocci in the family *Staphylococcaceae*, order *Bacillales*, and class *Bacilli*. Figure 1 shows the phylogenetic position of the GV69 in relation to other *Staphylococcus* spp. The GV69 strain is a facultatively anaerobic, mesophilic bacterium that can grow at temperatures of 30–37 °C. *S. aureus* isolates exhibit a preference for glycolytic carbon sources. Acid is produced aerobically and anaerobically from glucose, lactose, maltose and mannitol, and aerobically from fructose, galactose, mannose, ribose, sucrose, trehalose, turanose and glycerol [9]. Figure 2 shows a photomicrograph of the *S. aureus* GV69 strain using Gram stain technique.

GV69 cultures were grown at 37 °C with aeration (250 rpm) in rich media (tripticase soy broth) for 18 h, and the strain was initially identified by routine diagnostics based on Gram stain, mannitol fermentation, catalase testing and tube coagulase testing. A summary of the general information gathered for the GV69 is listed in the Table 1. Data from antimicrobial disc susceptibility test demonstrated that, in addition to methicillin and other β-lactam drugs, this strain is resistant to several different groups of antimicrobial drugs, although vancomycin and the more recent commercially available antibiotics are exceptions. In addition, GV69 strain shows an average biofilm unit of 0.86 (moderate biofilm phenotype), whereas BMB9393 has an average BU of 3.7 (strong biofilm phenotype) [6]. This strain was deposited at the public collection “Coleção de Micro-organismos de Referência em Vigilância Sanitária” of the Fundação Oswaldo Cruz with the reference name P4521 [10].

**Table 1:** Classification and general features of the methicillin-resistant *Staphylococcus aureus* GV69 strain [11]

MIGS ID	Property	Term	Evidence code^a^
	Current classification	Domain *Bacteria*	TAS [24]
		Phylum *Firmicutes*	TAS [9, 25–27]
		Class *Bacilli*	TAS [28, 29]
		Order *Bacillales*	TAS [26–29]
		Family *Staphylococcaceae*	TAS [29, 30]
		Genus *Staphylococcus*	TAS [13, 30]
		Species *Staphylococcus aureus*	TAS [13, 30]
		Type strain MRSA GV69	IDA
	Gram stain	Positive	TAS [30]
	Cell shape	Coccus	TAS [30]
	Motility	Non-motile	TAS [30]
	Sporulation	Non-sporulating	NAS
	Temperature range	Mesophile	TAS [30]
	Optimum temperature	30–37 °C	TAS [30]
	pH range; Optimum	4.2-9.3; 7.0-7.5	TAS [9]
	Carbon source	Glucose, lactose, maltose and mannitol	TAS [9]
GS-6	Habitat	Skin wound	IDA
MIGS-6.3	Salinity	7.5 %	TAS [30]
MIGS-22	Oxygen requirement	Facultative anaerobe	TAS [30]
MIGS-15	Biotic relationship	Commensal	TAS [30]
MIGS-14	Pathogenicity	Opportunistic pathogen	NAS
MIGS-4	Geographic location	Teresina, PI, Brazil	IDA
MIGS-5	Sample collection	June 1996	IDA
MIGS-4.1	Latitude	05° 05’ 21” S	IDA
MIGS-4.2	Longitude	42° 48’ 07” W	IDA
MIGS-4.4	Altitude	72 m	IDA

^a^ Evidence codes, *IDA* Inferred from Direct Assay, *TAS* Traceable Author Statement (i.e., a direct report exists in the literature), *NAS* Non-traceable Author Statement (i.e., not directly observed for the living, isolated sample, but based on a generally accepted property for the species, or anecdotal evidence). These evidence codes are from the Gene Ontology project [19]

## Genome sequencing information

### Genome project history

A collaboration between the Laboratório Nacional de Computação Científica, operated by the Ministério de Ciência e Tecnologia e Inovação of the Brazilian government, and the Universidade Federal do Rio de Janeiro sequenced, assembled, and annotated the complete GV69 genome as part of the ST239 Genome Program. This organism was selected for sequencing as a representative of the approximately 30 % of Brazilian ST239 isolates that display an *agr* dysfunction. The raw sequence data was deposited in NCBI’s Sequence Read Archive (experiment accession number SRX1322312 and GV69 run accession number SRR2601051). The complete genome sequence of the GV69 strain was deposited in GenBank (accession number CP009681). Table 2 presents the project information and its association with MIGS version 2.0 compliance [11].

**Table 2:** Project information

MIGS ID	Property	Term
MIGS 31	Finishing quality	Finished
MIGS 28	Libraries used	454 GS FLX 3-kb paired-end library
MIGS 29	Sequencing platforms	454 GS FLX Titanium
MIGS 31.2	Fold coverage	23×
MIGS 30	Assemblers	Newbler v2.6, Celera Assembler v6.1
MIGS 32	Gene calling method	Glimmer and GeneMark
	Locus Tag	SAGV69
	Genbank ID	CP009681
	Genbank Date of Release	October 28, 2014
	GOLD ID	Gp0108938
	BIOPROJECT	PRJNA264181
MIGS 13	Source Material Identifier	P4521
	Project relevance	Medical

### Growth conditions and genomic DNA preparation

A volume of 0.5 mL of a GV69 culture (37 °C/18 h) was inoculated into a 250 mL-Erlenmeyer flask containing 50 mL of pre-sterilized TSB. The culture was grown at 37 °C for 18 h under normal atmospheric conditions and shaking at 250 RPM. The bacteria were harvested by centrifugation (1500 × g at 4 °C), washed twice in cold sterile water and the whole pellet used for DNA preparation. Cells were lysed with 20U/mL lysostaphin and DNA obtained by phenol extraction and ethanol precipitation [12]. The concentration and purity of the resulting DNA was assessed using a Qubit® 2.0 fluorometer (Invitrogen; Eugene, Oregon, USA). This genomic DNA (5 μg) was used to prepare a paired-end library.

### Genome sequencing and assembly

The genome sequencing was performed using a 454 GS FLX Titanium (3-kb paired-end library) approach (Roche Diagnostics Corporation, Indianapolis, IN, USA). The assembly, based on 362,284 reads that corresponded to 62,981,906 bp (23-fold coverage), was performed using Newbler v2.6 (Roche) and Celera Assembler v6.1 [13]. Gaps within scaffolds resulting from repetitive sequences were resolved by *in silico* gap filling. For determining the small insertions and deletions occurring into homopolymer regions (at least three consecutive equal base pairs), the complete genomic sequence of the GV69 isolate was compared to that of the ST239 isolate, TW20, from United Kingdom, whose complete sequence is deposited in the GenBank (accession number: FN433596). For this comparison we applied Crossmatch (version 0.990329) with more stringent default parameter (mismatch = 14). The result of the alignment showed 541 inserts (of which 174 occurring into homopolymeric regions) and 575 deletes (of which 244 occurring into homopolymeric regions). In summary, the complete genome sequence of the GV69 isolate harbors 418 InDels occurring into homopolymer regions in relation to the genome sequence of the TW20 (Additional file 1: Table S1).

### Genome annotation

The genome annotation was performed using the System for Automated Bacterial Integrated Annotation [14]. This software uses an automated annotation pipeline, where each open reading frame is submitted to comparison with several databases (NCBI-nr, KEGG, InterPro and UniProtKB/Swiss-Prot), and the results are made available on the screen for the assessment of expert users*.* All possible ORFs are predicted by Glimmer [15] and GeneMark [16] and tRNAs are detected by tRNAscan-SE [17]. The identification of bona fide ORFs and their probable functions takes in account the results of similarity searches using both nucleotide and amino acid sequences by BLAST against KEGG, NCBI-nr and UniProtKB/Swiss-Prot databases, and also the prediction of protein domains and important sites using InterPro [18]. ORFs with a good BLAST coverage in the NCBI-nr database, with a minimum of 60 % positive identity, 80 % query coverage, 80 % subject coverage, and 10^−5^*e*-value cutoff were assigned as “valid”, with known function or hypothetical. On the other hand, when identified truncated version of a gene, because of nonsense or frameshift mutations in the coding sequence, the corresponding ORF was annotated as pseudogene. In addition, other analyses using SABIA pipeline comprised the classification of gene products according with biological processes, cellular components and molecular functions based on Gene Ontology [19, 20]. The functional classification according with biological systems was based on KEGG and COG databases. The identification and classification of membrane transport proteins was based on Transporter Classification system available in TCDB database, and subcellular localization of proteins was predicted using PSORT tool [21]. CRISPRFinder was used for identifying clustered regularly interspaced short palindromic repeats [22].

## Genome properties

**Fig. 3:**
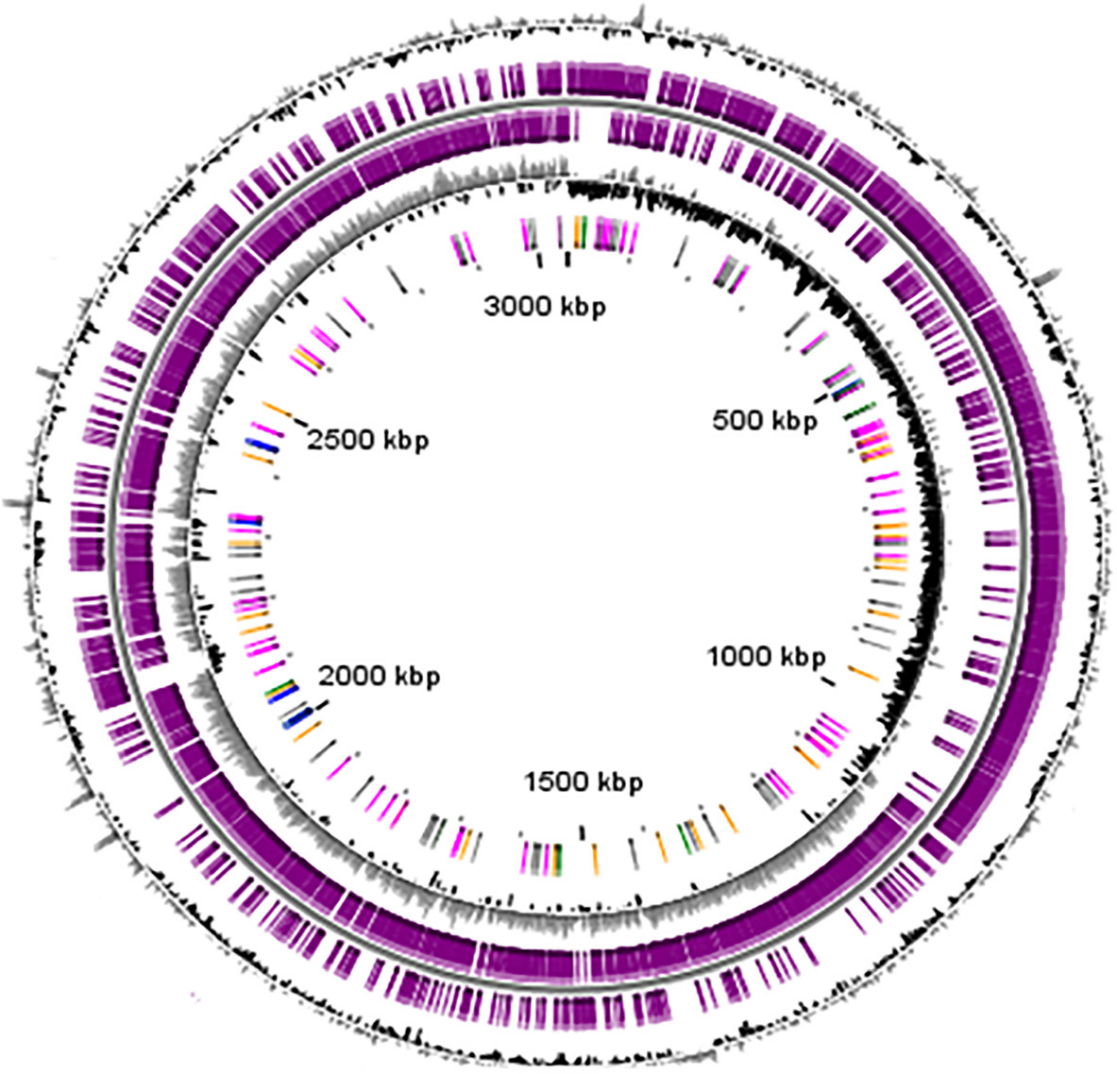
Circular representation of the *S. aureus* GV69. Circles display (from the outside): (1) GC percent deviation (GC window - mean GC) in a 1000-bp window; (2) Predicted CDSs transcribed in the clockwise direction; (3) Predicted CDSs transcribed in the counterclockwise direction- *Genes displayed in (2) and (3) are color-coded according different categories:* red and blue: MaGe validated annotations, orange: MicroScope automatic annotation with a reference genome, purple: Primary/Automatic annotations; (4) GC skew (G + C/G-C) in a 1000-bp window; (5) rRNA (blue), tRNA (green), misc_RNA (orange), Transposable elements (pink) and pseudogenes (grey). Map was constructed using GCViewer [33]

The GV69 genome consists of one circular chromosome of 3,046,210 bp with a G + C content of 32.94 % (Fig. 3). Using the SABIA pipeline [14], we functionally annotated 2,758 protein-coding sequences of which 2,285 were assigned to known functions, with the remaining 473 assigned to unknown categories. Seventy-six were assigned as putative pseudogenes. The genome harbors 5 rRNA operons (5 copies of 16S rRNA, 5 of 23S rRNA, and 6 of 5S rRNA) and 60 tRNA genes, which were identified with RNAmmer [23] and tRNAscan-SE [17], respectively. This information is summarized in Table 3. A total of 2,098 genes were assigned to COG; a breakdown of their functional assignments is shown in Table 4.

**Table 3:** Nucleotide content and gene count levels of the methicillin-resistant *Staphylococcus aureus* GV69 strain genome

Attribute	Value	% of Total^a^
Genome size (bp)	3,046,210	100.00
DNA coding (bp)	2,457,378	80.67
DNA G + C (bp)	1,003,422	32.94
DNA scaffolds	1	100.00
Total genes	2,910	100.00
Protein coding genes	2,758	94.78
RNA genes	76	2.61
Pseudogenes	76	2.61
Genes in internal clusters	NA^b^	NA
Genes with function prediction	2,285	78.52
Genes assigned to COGs	2,098	72.10
Genes with Pfam domains	2,444	83.99
Genes with signal peptides	652	22.41
Genes with transmembrane helices	599	20.58
CRISPRs repeats^c^	0	0

^a^The total is based on either the size of the genome in base pairs or the total number of genes in the annotated genome. ^b^Not annotated. ^c^Confirmed CRISPRs repeats = 0 [22]

**Table 4:** Number of protein coding genes of the methicillin-resistant *Staphylococcus aureus* GV69 strain associated with the general COG functional categories

Code	Value	%age^a^	Description
J	146	5.29	Translation
A	0	0.00	RNA processing and modification
K	140	5.08	Transcription
L	169	6.13	Replication, recombination, and repair
B	0	0.00	Chromatin structure and dynamics
D	28	1.02	Cell cycle control, mitosis, and meiosis
Y	0	0.00	Nuclear structure
V	40	1.45	Defense mechanisms
T	44	1.60	Signal transduction mechanisms
M	107	3.88	Cell wall/membrane biogenesis
N	3	0.11	Cell motility
Z	0	0.00	Cytoskeleton
W	0	0.00	Extracellular structures
U	29	1.05	Intracellular trafficking and secretion
O	69	2.50	Posttranslational modification, protein turnover, chaperones
C	97	3.52	Energy production and conversion
G	139	5.04	Carbohydrate transport and metabolism
E	177	6.42	Amino acid transport and metabolism
F	71	2.57	Nucleotide transport and metabolism
H	95	3.44	Coenzyme transport and metabolism
I	53	1.92	Lipid transport and metabolism
P	145	5.26	Inorganic ion transport and metabolism
Q	31	1.12	Secondary metabolite biosynthesis, transport, and catabolism
R	287	10.41	General function prediction only
S	228	8.27	Function unknown
-	660	23.93	Not in COGs

^a^The total is based on the total number of protein coding genes in the annotated genome

## Conclusions

Comparative analyses were performed using the SABIA pipeline [14]. The bidirectional best hit (90 % amino acid identity and 90 % alignment coverage) comparison with six other published ST239 *S. aureus* genomes revealed that GV69 shares 2,415 CDS with BMB9393, another Brazilian ST239 variant; 2,328 CDS with strain JKD6008; 2,357 with strain TW20; 2,342 with strain T0131; 2,380 with Z172; and 2,290 with XN108. Despite that, GV69 has 170 unique CDS relative to the other six genomes, including an extra copy of a gene encoding a putative N-acetylmuramoyl-L-alanine amidase, an enzyme related to the bacterial cell autolytic function. This gene is located in a phage-associated mobile genetic element (phage-associated) inserted in the chromosome.

Although belonging to the same lineage and clonal type, strains GV69 and BMB9393 have differences in their flexible genomes. In addition to 343 CDS (150 of unknown function, including several related to MGEs) found exclusively in GV69, this strain lacks a small 2,908 bp plasmid found in BMB9393 that carries the *cat* gene, a determinant for chloramphenicol resistance.

## Additional file

Additional file 1: Table S1. InDels into homopolymeric regions of the GV69 complete genome sequence in comparison with TW20 complete genome. (XLS 48 kb)

## Abbreviations

Agr: accessory gene regulator
BBH: bidirectional best hit
BEC: Brazilian epidemic clone
BU: biofilm unit
HA-MRSA: hospital-acquired methicillin-resistant *Staphylococcus aureus*
MGE: mobile genetic element
MLST: multilocus sequence typing
SABIA: system for automated bacterial integrated annotation
SCC*mec*: staphylococcal cassette chromosome *mec*
SGP: ST239 genome program
ST: sequence type
